# Numerical Study of Lid-Driven Hybrid Nanofluid Flow in a Corrugated Porous Cavity in the Presence of Magnetic Field

**DOI:** 10.3390/nano12142390

**Published:** 2022-07-13

**Authors:** Apichit Maneengam, Tarek Bouzennada, Aissa Abderrahmane, Kamel Guedri, Wajaree Weera, Obai Younis, Belgacem Bouallegue

**Affiliations:** 1 Department of Mechanical Engineering Technology, College of Industrial Technology, King Mongkut’s University of Technology North Bangkok, Bangkok 10800, Thailand; apichit.m@cit.kmutnb.ac.th; 2 Mechanics of Materials & Plant Maintenance Research Laboratory (LR3MI), Mechanical Engineering Department, Faculty of Engineering, Badji Mokhtar University, P.O. Box 12, Annaba 23052, Algeria; tarek.bouzennada@univ-annaba.org; 3 Laboratoire de Physique Quantique de la Matière et Modélisation Mathématique (LPQ3M), University of Mascara, Mascara 29000, Algeria; a.aissa@univ-mascara.dz; 4 Mechanical Engineering Department, College of Engineering and Islamic Architecture, Umm Al-Qura University, P.O. Box 5555, Makkah 21955, Saudi Arabia; kmguedri@uqu.edu.sa; 5 Department of Mathematics, Faculty of Science, Khon Kaen University, Khon Kaen 40002, Thailand; 6 Department of Mechanical Engineering, College of Engineering at Wadi Addwaser, Prince Sattam Bin Abdulaziz University, Wadi Addwaser 11911, Saudi Arabia; oubeytaha@hotmail.com; 7 College of Computer Science, King Khalid University, Abha 61413, Saudi Arabia; bbelgacem@kku.edu.sa; 8 Electronics and Micro-Electronics Laboratory, Faculty of Sciences of Monastir, University of Monastir, Monastir 1002, Tunisia

**Keywords:** mixed convection, entropy production, hybrid nanofluid, wavy wall

## Abstract

The lid-driven top wall’s influence combined with the side walls’ waviness map induce the mixed convection heat transfer, flow behavior, and entropy generation of a hybrid nanofluid (Fe_3_O_4_–MWCNT/water), a process analyzed through the present study. The working fluid occupies a permeable cubic chamber and is subjected to a magnetic field. The governing equations are solved by employing the GFEM method. The results show that the magnetic force significantly affects the working fluid’s thermal and flow behavior, where the magnetic force’s perpendicular direction remarkably improves the thermal distribution at Re = 500. Also, increasing Ha and decreasing Re drops both the irreversibility and the heat transfer rate. In addition, the highest undulation number on the wavy-sided walls gives the best heat transfer rate and the highest irreversibility.

## 1. Introduction

The dispersion of nanoparticles in standard cooling liquids has garnered substantial interest in recent years both in industrial and academic fields. The blend which results from this mixing is termed the nano-liquid or nanofluid. This technology of smart liquids was effectively exploited in numerous actual applications such as in renewable energy equipment, air-conditioning, transportation, lubricating oils, micro-manufacturing, and automobile cooling systems [1,2,3,4]. Currently, the expansion of intensification techniques for energy transmission in various appliances represents an intriguing task in several industrial disciplines.

The thermal transmission in nanofluids may be controlled by incorporating moving surfaces and local heaters, and applying external forces such as magnetic fields [5,6,7,8]. Mixed convection motion heat transfer in nanofluids within diverse containers involving sliding walls has numerous technical uses. Examination of such an issue is vital in managing the fluid motion and energy transfer for solar absorber tubes and thermal energy storage applications and boosting the performance of electronic cooling and nuclear devices.

Celik et al. [9] numerically investigated the heat transfer enhancement within a vertical channel, where a nanofluid passes through. The results showed that the heat transfer rate would enhance by increasing the Reynolds and Richardson numbers and adding 5% of nanoparticles.

Hasan et al. [10] proposed a numerical study to predict the flow and thermal behaviors of a fluid inside a rotating curved square. The authors found that the fluid is considerably mixed as the rotation rate of the curved enclosure increases, as a result, the heat transfer is noticeably enhanced. Darzi et al. [11] provided 3D and 2D numerical investigations to illustrate the influence of Cu-based nano-liquid on mixed convection in a sliding enclosure employing the two-phase mixture model. Their outcomes show that the usage of fines failed to augment the heat transfer near modest and minimal Richardson values due to the forming of the motion obstruction within the corners of these introduced extended surfaces. The impacts of a sliding wall on mixed convection in a chamber with an undulating wall saturated with a nano-liquid have been numerically analyzed by Abu-Nada et al. [12]. They discovered that increasing the volume percentage of nanoparticles enhances the heat transfer for all levels of lower wall geometry ratios and Ri numbers. Alsabery et al. [13] examined the mixed convective heat transfer inside a chamber filled with a nano-liquid. Their simulation includes the influences of centered heat-conducting cylinder, cold surface mobility, and local heating. A nonlinear influence of nanoparticle concentration on the heat transfer rate for high Reynolds number has been established. Aljabair et al. [14] presented a numerical study of the convective heat and mass transfer of Cu–H_2_O nano-liquid inside an arc chamber with inconsistent heating and a top flat sliding wall. Their findings suggest that the sliding baffle becomes more active to boost heat transfer. Therefore, non-uniform heating and arc cavities are suitable instances to boost thermal energy transfer. Louaraychi et al. [15] quantitatively examined mixed heat transfer in a chamber with two moving walls. They also employed the SIMPLER approach for linking velocity and pressure equations and demonstrated that the transition from one dominated regime to another depended on the ratio of Ra/Pe.

The magnetic field may be employed as a passive strategy to control heat transfer and liquid motion for sliding chambers [16,17,18,19]. Ali et al. [20] studied the phenomenon of mixed convection in a sliding chamber. Their findings revealed that hybrid nano-liquid and the increase of Richardson (*Ri*) number produce an increase in the heat transfer rate, however, it decreased for big chamber length. Oztop et al. [21] explored mixed convection motion incorporating the magnetic field into a sliding container heated at the edge. They proved that the magnetic force significantly affects the management the heat and liquid development. In opposition, the free convection prevailing regime is crucially influenced by the characteristics of the heater. In another work [22], they investigated mixed convection heat transfer and liquid motion in a lid-driven square chamber with a circular core. Their experimental results demonstrated that the circular body may be a controlling factor for heat and liquid flow. Garmroodi et al. [23] used the FVM to computationally model the MHD mixed convention within a sliding enclosure. The Nu declined with the growing Ha number for all studied configurations of the spinning cylinders. Nath et al. [18] explored the effects of nanoparticles’ form on heat and mass transport processes in a moving lid chamber under the combined action of thermo-solutal buoyant force and magnetic field. The findings indicated that the Nusselt number is considerably dependent upon the tilted magnetic force and buoyancy ratio, however, on mass transfer the shape influence is minimal. Sheikholeslami et al. [24] explored the effect of a changeable magnetic domain on the features of mixed convection motion inside the moving lid enclosure containing undulated hot plans. It was found that an increase in the heat transfer was obtained by increasing *Re* number, nanoparticle concentration, or magnetic intensity, however, the inverse occurs when *Ha* number increase.

Many researchers investigated mixed convection in a permeable medium under a variety of settings, such as lid-driven cavities filled with nano-liquid. Abu-Hamdeh et al. [25] created an in-house code to investigate mixed convective heat transfer in a moving lid enclosure where one of the sides is an opening wall saturated by permeable media. They reported that heat transfer rate increases when Gr and heater length increase, however, it drops with increasing *Da*. Ahmed et al. [26] evaluated the influence of magnetic forces inclination and double moving lid on the mixed convection in a rectangular chamber loaded with a Cu–H_2_O micropolar nano-liquid saturated permeable material. Syed S. Shah et al. [27] developed a computer model for mixed convection moving lid motion of CuO–H_2_O nano-liquid confined in a curved wavy chamber with cylindrical obstacles with three distinct conditions. The findings suggested that *Nu* rises for high *Da* due to the convection in the lid chamber. For high *Re*, normally *Nu* falls or maintains the same value at the wall with the growth of nanoparticles in permeable media. Ahmed et al. [28] explored entropy production owing to varied factors in a porous lid-driven cavity under the mixed convective MHD flow. The heater in this research is positioned in the right corner of the chamber and they noticed that the right side of the heater has a greater influence on the entropy production and a drop in Darcy number produces a reduction in heat transfer. Marzougui et al. [16] concentrated on entropy study in a permeable moving lid chamber controlled by Lorentz force and occupied with Cu–H_2_O nano-liquid. Although the magnetic force was provided evenly, the magnetic field, dependent on motion velocity, fluctuate with its inclination angle, and thus every 30° the variation in magnetic entropy production is noticed.

For of its vast applicability in various engineering challenges such as solar collectors, microelectronic devices, and so on, studies dealing with convective heat transmission domains inside complicated geometries such as cavities with wavy walls, various studies in the literature reported that the heat transfer efficiency relies on the precise values applied to the geometrical features of the undulating surface. Al-Amiri et al. [29] examined the mixed convection in a moving lid chamber with a waveform heated lower. They evaluated the impacts of *Ri*, and undulation wall on motion pattern and heat transmission properties. The influences of various parameters on the buoyant convection of a permeable chamber heated partly at the lower wall have been investigated by Alsabery et al. [30] They observed that heat transmission is greatly impacted by the length and location of the heater. Rashed et al. [31] examined the radiation impact of MHD heat transfer of Cu–Al_2_O_3_ nano-liquid in an angled chamber with cold undulating side plans and partial heating at the upper and lower side with internal heat production. They observed that the undulation number, cavity orientation, and radiation condition have a major effect on the thermal efficiency of the chamber. Hatami et al. [32] have used a computational and statistical technique to discover the optimal undulating profile for the lower surface of a nano-liquid-based direct PTSC. Their findings show that the optimum option is to choose a modest mean oscillation number and amplitude for the undulating wall. Mansour et al. [33] examined the heat transmission in a double-isothermal walled chamber packed with a nano-liquid. It has been demonstrated that the heat transmission efficiency may be adjusted by altering the wavy plans. Lin et al. [34] analyzed the influence of various Prandtl number liquids within wavy permeable chambers. They observed that thermal energy transport is higher as the active duration of heating rises. Alsabery et al. [35] evaluated the influence of a two-phase nano-liquid method on mixed convection characteristics within a wavy-walled hollow holding a square solid block. They suggested that the temperature drop might generate a considerable concentration fluctuation in the chamber. Furthermore, the position of the solid block and undulation of wall plans are beneficial in the regulation of the heat transmission and the concentration of solid nanoparticles. Nirmalendu et al. [36] investigated the influence of partly active Lorentz force on the increased thermal efficiency of nano-liquid (Cu–Al_2_O_3_–H_2_O) inside a porous oblique-wavy chamber heated partially. They revealed that the existence of a complex wavy surface boosts the heat transfer by ~22.16% in comparison to a simple vertical wall; nevertheless, the intensity of liquid circulation diminishes. Aly et al. [37] explored the thermo-hydraulic behavior of a Cu–H_2_O-occupied permeable chamber with both wavy sides and heated differently. Their results show that the presence of nanoparticles enhances heat transfer.

Finally, simulation work has been performed by Boudraa and Bessaïh [38] to analyze the heat and entropy development of a nanofluid passing a channel that contains hot blocks placed on the bottom wall. The turbulent force convection was the governing heat transfer mode. The outcomes depicted that the heat dissipation and total entropy development are directly related to Reynolds and the nanoparticles’ weight.

Also, a competition analysis by Kumar et al. [39] led to summarize that the heat irreversibility extends with the increase of the nanoparticles concentration in a micro-channel, which permits a nanofluid to pass through.

Consequently; the lack of comprehension of the influence of the lid-driven wall and Lorentz force on the mixed convection flow of hybrid nanofluids especially within 3D-complexed porous designs is an encouraging factor to conduct the present study; whereas; this study is a theoretical proposal aiming to shed light on how the heat, flow, and entropy of hybrid nanofluids would be affected inside a 3D-complexed porous configuration in the presence of a sliding top baffle exposed to a magnetic field. However, the findings of this theoretical work would assist in developing the heat evacuation systems in many engineering domains such as wavy condensers of refrigerators [40], solar central receivers, nuclear power agitators, and cooling electronic elements [41].

## 2. Mathematical Model

**Figure 1:**
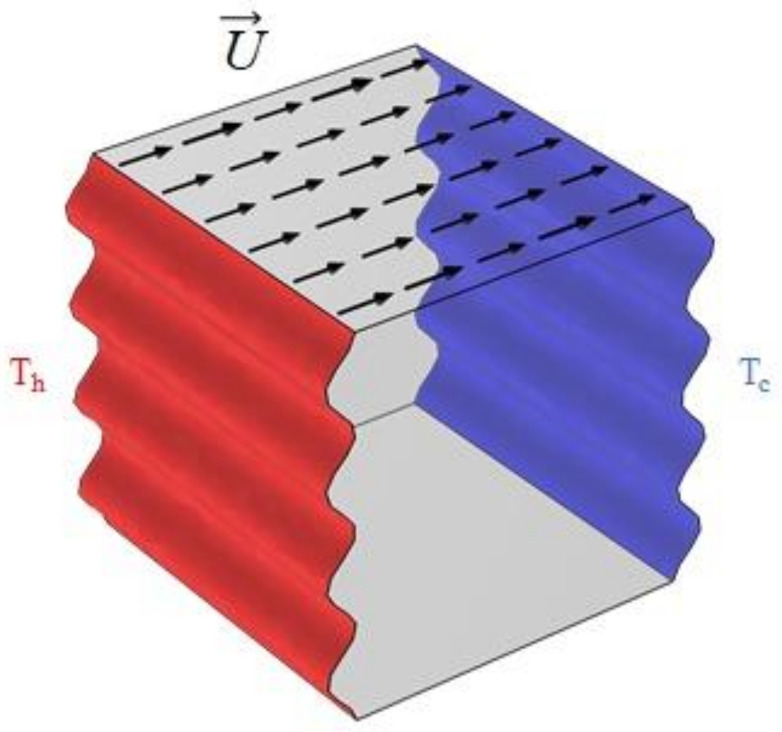
The computational domain and the boundary conditions.

The selected hybrid nanofluid consists of water, Fe_3_O_4_, and MWCNT where their thermophysical properties are arranged in Table 1. The physical configuration corresponds to a 3D lid-driven porous cavity filled with a nano-liquid where a magnetic field is applied perpendicular to the direction of the gravitational force (Figure 1). It imposes that all boundaries are stationary and non-slip except for two wavy sidewalls, where one is hot and the other is cold and maintained at temperatures *T_h_* and *T_c_*, respectively. Furthermore, the upper wall moves in the opposite direction at a constant speed, *U*. The wavy walls will have various patterns (different peak numbers, *N* = 4, 3, 2, and 1) because they are considered the configuration influencer.

The governing equations are as follows [44], assuming the investigation is conducted within a 3D porous cavity and the chosen liquid is a Newtonian-incompressible fluid in the laminar regime.

(1) $$\frac{\partial U}{\partial X}+\frac{\partial V}{\partial Y\;}+\frac{\partial W}{\partial Z}=0$$

(2) $$\frac{\rho_{hnf}}{\rho_{f}}\left[\frac{U}{\varepsilon^{2}}\frac{\partial U}{\partial X}+\frac{V}{\varepsilon^{2}}\frac{\partial U}{\partial Y}+\frac{W}{\varepsilon^{2}}\frac{\partial U}{\partial Z}\right]\;\;=-\frac{\rho_{hnf}}{\rho_{f}}\frac{\partial P}{\partial X}+\frac{1}{Re}\frac{1}{\varepsilon }\frac{\mu_{hnf}}{\mu_{f}}\left(\frac{\partial U}{\partial X}+\frac{\partial U}{\partial Y}+\frac{\partial U}{\partial Z}\right)-\frac{\mu_{hnf}}{\mu_{f}ReDa}U\;-\frac{\rho_{hnf}}{\rho_{f}}\frac{0.55}{\sqrt{Da}}\sqrt{U^{2}+V^{2}+W^{2}}\;U\;$$

(3) $$\frac{\rho_{hnf}}{\rho_{f}}\left[\frac{U}{\varepsilon^{2}}\frac{\partial V}{\partial X}+\frac{V}{\varepsilon^{2}}\frac{\partial V}{\partial Y}+\frac{W}{\varepsilon^{2}}\frac{\partial V}{\partial Z}\right]\;\;=-\frac{\rho_{hnf}}{\rho_{f}}\frac{\partial P}{\partial Y}+\frac{1}{Re}\frac{1}{\varepsilon }\frac{\mu_{hnf}}{\mu_{f}}\left(\frac{\partial V}{\partial X}+\frac{\partial V}{\partial Y}+\frac{\partial V}{\partial Z}\right)-\frac{\mu_{hnf}}{\mu_{f}ReDa}V\;-\frac{\rho_{hnf}}{\rho_{f}}\frac{0.55}{\sqrt{Da}}\sqrt{U^{2}+V^{2}+W^{2}}V-\frac{\sigma_{hnf}}{\sigma_{f}}Ha^{2}\;\frac{V}{\varepsilon }\;$$

(4) $$\frac{\rho_{hnf}}{\rho_{f}}\left[\frac{U}{\varepsilon^{2}}\frac{\partial W}{\partial X}+\frac{V}{\varepsilon^{2}}\frac{\partial W}{\partial Y}+\frac{W}{\varepsilon^{2}}\frac{\partial W}{\partial Z}\right]\;\;=-\frac{\rho_{hnf}}{\rho_{f}}\frac{\partial P}{\partial Z}+\frac{1}{Re}\frac{1}{\varepsilon }\frac{\mu_{hnf}}{\mu_{f}}\left(\frac{\partial W}{\partial X}+\frac{\partial W}{\partial Y}+\frac{\partial W}{\partial Z}\right)-\frac{\mu_{hnf}}{\mu_{f}ReDa}W\;-\frac{\rho_{hnf}}{\rho_{f}}\frac{0.55}{\sqrt{Da}}\sqrt{U^{2}+V^{2}+W^{2}}\;W+\frac{{\left(\rho \beta \right)}_{hnf}}{{\left(\rho \beta \right)}_{f}}\frac{Gr}{Re^{2}}\theta -\frac{\sigma_{nf}}{\sigma_{f}}Ha^{2}\;\frac{W}{\varepsilon }$$

(5) $$U\frac{\partial \theta }{\partial X}+V\frac{\partial \theta }{\partial Y}+W\frac{\partial \theta }{\partial Z}=\frac{{\left(\rho c_{p}\right)}_{f}}{{\left(\rho c_{p}\right)}_{hnf}}\frac{k_{hnf}}{k_{f}}\frac{1}{RePr}\left[\frac{\partial^{2}\theta }{\partial X^{2}}+\frac{\partial^{2}\theta }{\partial Y^{2}}+\frac{\partial^{2}\theta }{\partial Z^{2}}\right]$$

where $X,Y,Z=\frac{x,y,z}{L}$, $U,V,W=\frac{\left(u,v,w\right)L}{\alpha_{f}}$, $\theta =\frac{T-T_{c}}{T_{h}-T_{c}}$, $P=\frac{pL^{2}}{\rho_{f}\alpha_{f}^{2}}$, $\mathrm{Pr}=\frac{v_{f}}{\alpha_{f}}$, $Da=\frac{K}{L^{2}}$$Ra=\frac{g\beta_{f}(T_{h}-T_{c})L^{3}}{\alpha_{f}v_{f}}$, $Ha=LB\sqrt{\frac{\sigma_{hnf}}{\mu_{hnf}}}$, ε is the porosity, $Ri=\frac{Gr}{Re^{2}}$.

Designed for nanoparticles Fe_3_O_4_–MWCNT, the properties are arranged in Table 2.

The total entropy may be characterized as follows [45]:

(6) $$S_{tot}=S_{ht}+S_{ff}+S_{mf}$$

(7) $$S_{ht}=\frac{k_{hnf}}{T_{0}^{2}}\left[{\left(\frac{\partial T}{\partial x}\right)}^{2}+{\left(\frac{\partial T}{\partial y}\right)}^{2}+{\left(\frac{\partial T}{\partial z}\right)}^{2}\right]$$

(8) $$S_{ff}=\frac{\mu_{hnf}}{T_{0}}[2\left({\left(\frac{\partial u}{\partial x}\right)}^{2}+{\left(\frac{\partial v}{\partial Y}\right)}^{2}+{\left(\frac{\partial w}{\partial z}\right)}^{2}\right)+{\left(\frac{\partial u}{\partial y}+\frac{\partial v}{\partial x}\right)}^{2}+{\left(\frac{\partial w}{\partial y}+\frac{\partial v}{\partial z}\right)}^{2}\;+{\left(\frac{\partial u}{\partial z}+\frac{\partial w}{\partial x}\right)}^{2}]\;+\frac{\mu_{hnf}}{T_{0}K}\left(u^{2}+v^{2}+w^{2}\right)\;$$

(9) $$S_{mf}=\frac{\sigma_{hnf}}{T_{0}}{\left(v\times B_{0}\right)}^{2}$$

with $T_{0}=\frac{T_{C}+T_{h}}{2}$.

Entropy generation Stot in non-dimensional form read:

(10) $$S_{TOT}=S_{HT}+S_{FF}+S_{MF}$$

(11) $$S_{HT}=\frac{k_{hnf}}{k_{f}}\left[{\left(\frac{\partial \theta }{\partial X}\right)}^{2}+{\left(\frac{\partial \theta }{\partial Y}\right)}^{2}+{\left(\frac{\partial \theta }{\partial Z}\right)}^{2}\right]$$

(12) $$S_{FF}=\frac{\mu_{hnf}}{\mu_{f}}N_{\mu }\left\{\begin{array}{c} \left[2{\left(\frac{\partial U}{\partial X}\right)}^{2}+2{\left(\frac{\partial V}{\partial Y}\right)}^{2}+2{\left(\frac{\partial W}{\partial Z}\right)}^{2}\right]\\ +{\left(\frac{\partial^{2}U}{\partial Y^{2}}+\frac{\partial^{2}V}{\partial X^{2}}\right)}^{2}+{\left(\frac{\partial^{2}W}{\partial Y^{2}}+\frac{\partial^{2}V}{\partial Z^{2}}\right)}^{2}+{\left(\frac{\partial^{2}U}{\partial Z^{2}}+\frac{\partial^{2}W}{\partial X^{2}}\right)}^{2} \end{array}\right\}\;+\frac{\mu_{hnf}}{\mu_{f}}N_{\mu }\left(\frac{U^{2}+V^{2}+W^{2}}{Da}\right)\;$$

(13) $$S_{MF}=N_{\mu }\frac{\sigma_{hnf}}{\sigma_{f}}Ha^{2}\left(U^{2}+V^{2}\right)$$

where $N_{\mu }=\frac{\varepsilon \mu_{hnf}T_{0}}{k_{hnf}}{\left(\frac{\alpha_{nf}}{L\Delta T}\right)}^{2}$.

The $Nu_{loc}$ and $Nu_{avg}$ are estimated as:

(14) $$Nu_{loc}=-\frac{k_{hnf}}{k_{f}}{\left(\frac{\partial \theta }{\partial n}\right)}_{wall},Nu_{avg}=\frac{1}{S^{2}}\int_{0}^{s}\int_{0}^{s}Nu_{loc}dydz$$

## 3. Numerical Method and Validation

Equations (1)–(5) are numerically treated utilizing the Galerkin weighted residual finite element approach [46,47,48]. This approach simplifies the solution of nonlinear partial differential equations by converting them to a set of integral equations. The computation cell is segmented by employing non-uniform triangular sections. The triangular sections with six nodes are used to generate the finite element equations. Following that, each term is decoded by employing Gauss’s quadrature approach.

The goal is to construct a collection of nonlinear algebraic equations that meet the boundary conditions. The computation processes stop when the relative error of each variable achieves the following convergence criterion:

(15) $$\left|\frac{\Gamma^{i+1}-\Gamma^{i}}{\Gamma^{i+1}}\right|\leq \eta$$

where *i* is the iteration value and *η* defines the convergence criterion, where *η* = 10^−6^.

**Figure 2:**
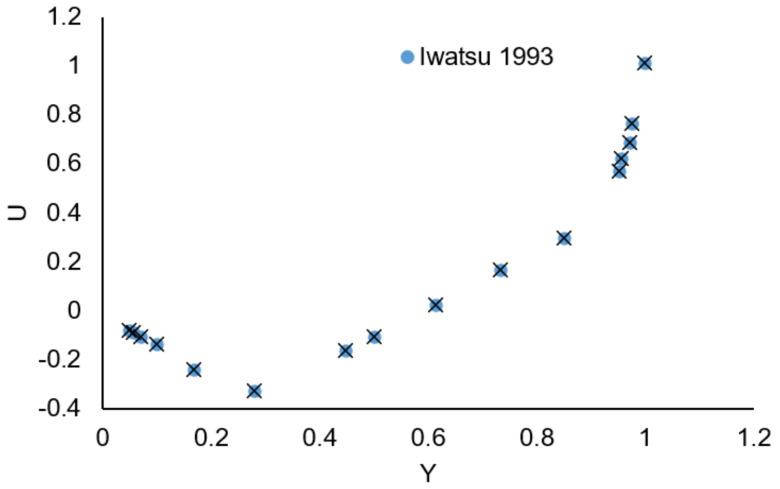
Comparison of present work with the work of Iwatsu et al. [49].

By testing with a few layouts, grid-independent solutions are identified. A grid of 480787 elements was employed to perform all simulations (see Table 3). To ensure that the numerical technique used to convert the authorized code is correct, the velocity profile inside the enclosure with obstructions is compared to the work of Iwatsu et al. [49] (see Figure 2).

## 4. Results and Discussion

Numerous studies have extensively discussed the effect of the percentage of additional nanoparticles, and almost all works concluded that a high concentration of nanoparticles boosts the thermal conductivity of the host fluid, meanwhile, a high concentration of these additives decreases the fluidity of the liquid. In most of these studies, the weight of the nanoparticles was limited to 6% [50], so in this study, the volume fraction of nanoparticles has been set at 0.04 to avoid any flow stagnation that might be affected by the high nano-concentration, and to benefit from the desirable role of these particles in heat transfer enhancement, while also focusing on the other influencer factors such as Da or Ha (it is well known that these two numbers affect the fluidity of the host fluid).

In the following section, the impacts of many factors on velocity pathlines, isothermal surfaces, and total entropy of the present hybrid nanofluid will be discussed in detail, where the velocity pathlines, isothermal surfaces, and total entropy outcomes are addressed, respectively, in the left, middle, and right columns.

The main influence parameters of the performed study are listed in the following Table 4.

**Figure 3:**
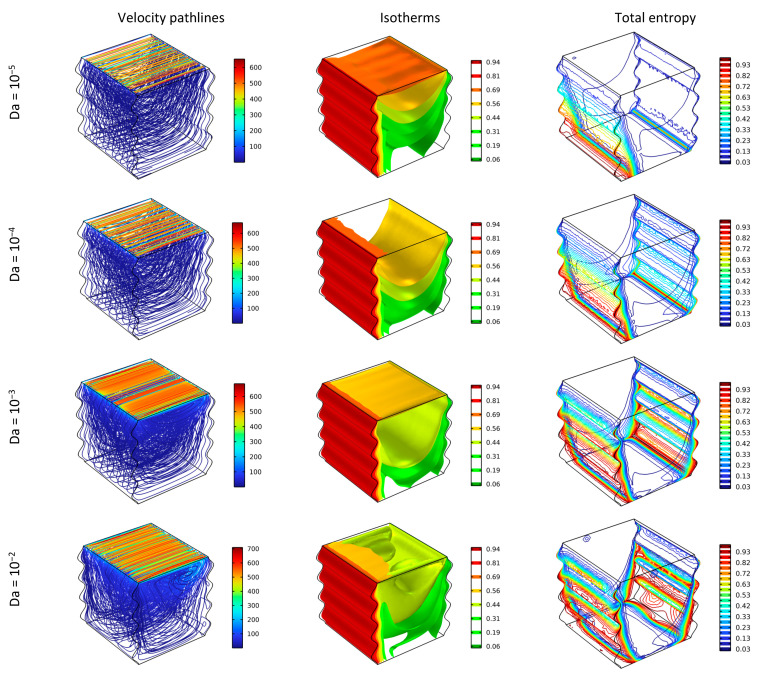
Distribution of the streamlines, isotherms, and total entropy for different Da for Ha = 0, φ = 0.04, and Re = 100.

Figure 3 illustrates the effect of different values of Da number ($Da\in \left[10^{-5},10^{-2}\right]$) for Re = 100 without the existence of a magnetic field, while adding 4% of nanoparticles on fluid flow, heat transfer, and total entropy.

Expanding the Darcy number makes the fluidity stronger and the values of the velocities higher, particularly in the closest layers to the top moving wall as in the streamlines display. In addition, the isothermal surfaces have a regular curvy shape downward with the most inferior value of Da, yet, irregular and random shapes starts to appear intensively in the inferior layers by augmenting Da. At the same time, heightening Da improves the heat distribution, where the high fluidity guides the heat to spread in a more efficient manner, and that can be marked from the upper isothermal surface. On the other hand, increasing Da drives the entropy to extend and spread widely over the cavity and achieve high values as depicted by the red lines. Da positively affects the heat transfer performance by advancing the fluid motion while at the same time increasing the fluid friction of the system, therefore, the total entropy production due to fluid friction and heat transfer builds up.

**Figure 4:**
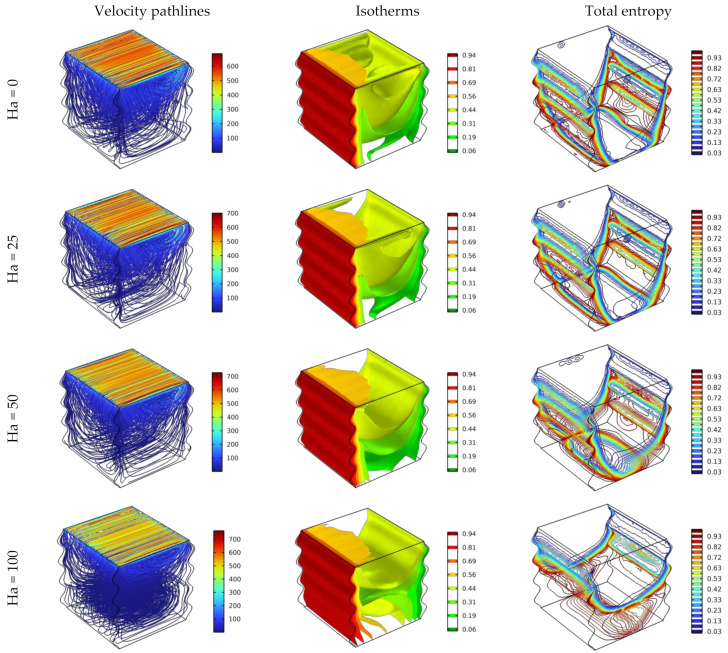
Distribution of the streamlines, isotherm, and total entropy for different Ha for Da = 10^–2^, φ = 0.04, and Re = 100.

The effect of applying various magnetic fields (Ha = 0, 25, 50, and 100) for Da = 0.01 and Re = 100 on pathlines, isothermal, and total entropy of 4% hybrid nanofluid is illustrated in Figure 4.

It can be observed that the fluid circulates around the y-direction due to the buoyancy force of free convection and the horizontal sliding action of the top wall for all cases of Hartman values, however, the fluidity intensity and the velocities of the fluid layers near the sliding top border diminish as the magnetic force increases. On the other hand, with regard to isothermal surfaces, it was detected that the heat transmits gradually from the hot wall to the front cold wall of all Hartman cases. The figure of the isotherms shows downwardly curving shapes, however, this can be explained by the liquid movement driving the heat to distribute in that shape, where that which refers to the convection is the dominant heat transfer mode.

On other hand, the simulations of the total entropy uncovered that increasing the Hartman number relieves the incidence of the generated entropy, particularly in the bottom area of the cavity.

**Figure 5:**
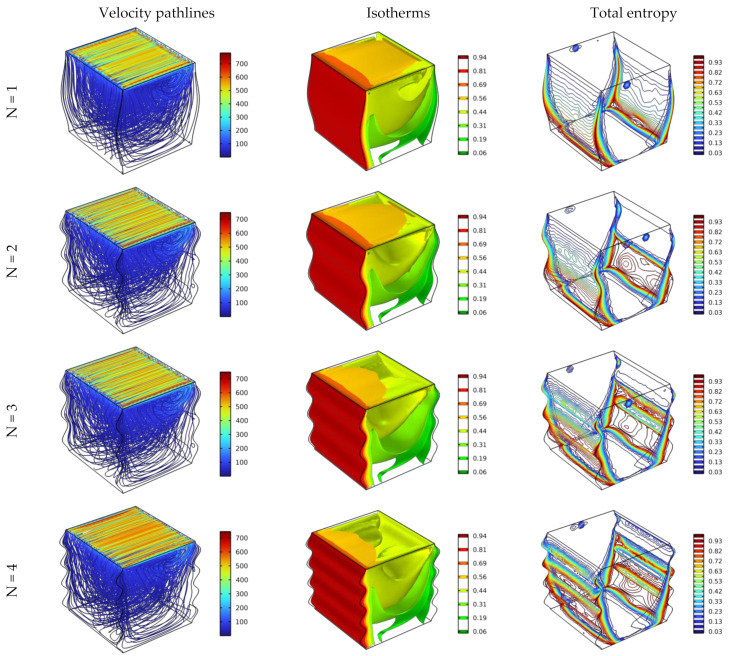
Distribution of streamlines, isotherm, and total entropy for Ha = 0, Da = 10^−2^, φ = 0.04, and Re = 100.

Figure 5 displays the effect of utilizing different wavy patterns (different peaks, N = 1, 2, 3, and 4) of the hot/cold side walls on flow behavior, heat transfer, and entropy for Da = 0.01 and Re = 100 with an addition of 4% of hybrid nano additives.

The pathlines results show a tiny change, where a slight increment in velocity can be observed near the top wall by enriching the undulation pattern of the two side walls (the hot/cold walls), and the same is found with the entropy development. Regarding the isothermal surfaces, it is shown that the heat transfer is improved by enriching the undulation of the wavy sidewalls, where this observation is based on the isothermal surface that is located parallel to the moving top wall, where this Iso-surface shrinks by increasing the peaks of the wavy walls. Furthermore, it can be suggested that the wavy wall might invigorate the fluid flow, so that enhances the heat transfer.

**Figure 6:**
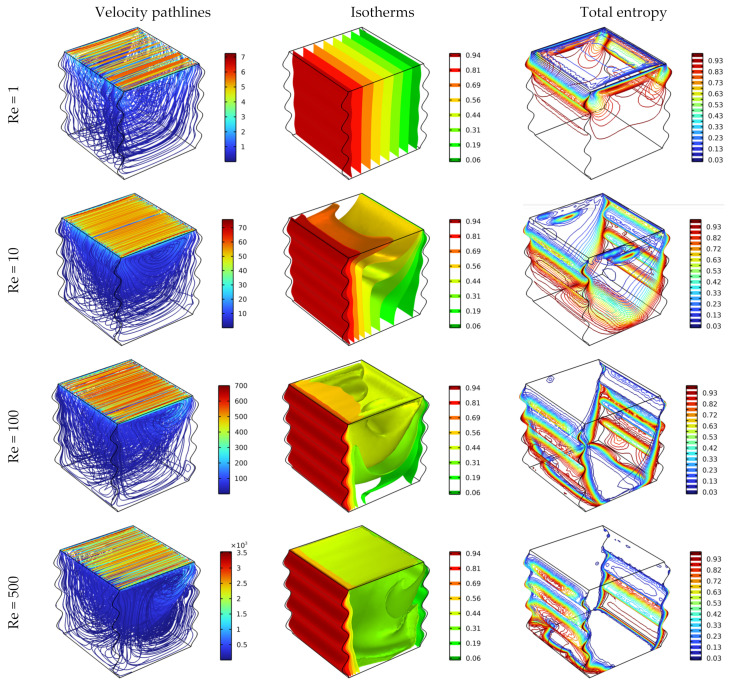
Distribution of streamlines, isotherm, and total entropy for different Re for Ha = 0, Da = 10^–2^, and φ = 0.04.

Figure 6 demonstrates the impact of the Reynolds number (Re = 1, 10, 100, and 500) on fluid flow, heat distribution, and total generated entropy, the Darcy number is fixed at 0.01 without applying magnetic force. The results of pathlines clarify that the strength of the fluid motion and the highest velocities are obtained by enlarging the Reynolds number, and that impacts the heat transfer performance, where the best heat transfer increases with Re, furthermore, it is observed that with the two highest values of Re, the isothermal surfaces form in random and irregular shapes. Whereas, with the case of Re = 1, the isothermal surfaces take a regular form, and that refers to the domination of the conduction mode. With the case of Re = 10, the isothermal surfaces take a slantwise shape, due to the effect of mixed convection, however, it can be said that the domination of the forced convection is desirable to improve the heat distribution, where the elevated values of Re make the fluid motion stronger, resulting in faster heat transfer. In another detail, high values of Re drive the total entropy to spread intensively, where the high fluid motion produces high fluid friction, so the total entropy (combined from entropy due to the fluid friction and heat transfer) increases.

**Figure 7:**
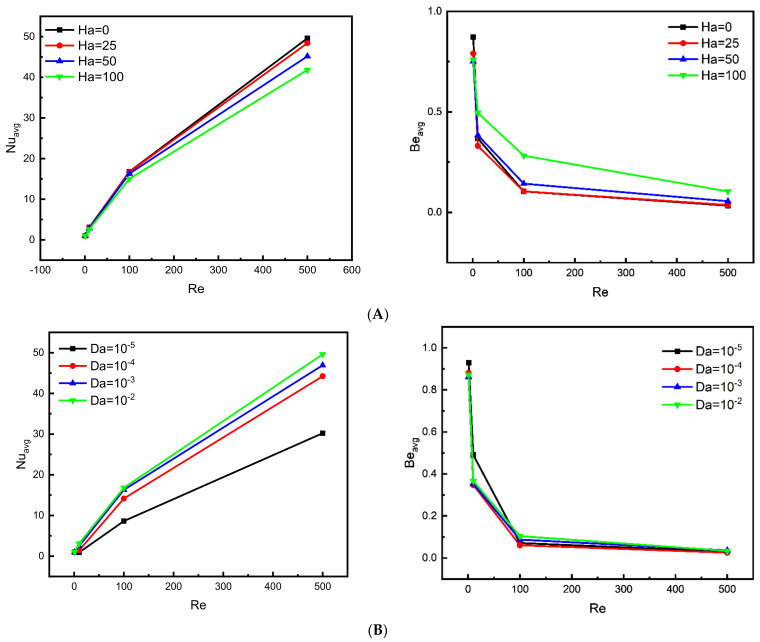
The mean Nusselt and Bejan (Be) development undergoing different values of Ha and Re for φ = 0.04 and Da = 0.01 (**A**), and undergoing different values of Da (**B**) and Re for φ = 0.04, Ha = 0.01.

Figure 7A present the evolutions of the Nu_avg_ and Bejan numbers when applying different values of Hartman and Reynolds.

The Nusselt number increases in line with raising the Re number or decreasing the Ha number. As it is known, the Nusselt number refers to the rate of heat dissipation, where the higher this number, the higher the rate of heat transfer. It is noted that a better heat transfer rate is obtained with the highest Re number (forced convection) and the closer the Ha number to zero, where the rate of heat transfer is related to fluid fluidity and the magnetic field inhibits the motion of the fluid and reduces the effectiveness of heat transfer by convection. Therefore, it is found that Nusselt’s highest values are with the case of Ha = 0/Re = 500.

In addition, it may be useful to recall that the Bejan number expresses the ratio of entropy caused by heat transfer over entropy due to the friction of the fluid layers during its movement.

It is found that there is a direct relationship between Bejan and Hartman’s numbers, however, regardless of the absence of a noticeable difference between the case of Ha = 0 and Ha = 25, they offer the lowest values in Bejan’s number. Likewise, Bejan’s number is noticed to take on lower values as the Reynolds number upgrades. It can be explained that with inferior values of the Ha number and elevated values of the Re number, the motion of the fluid becomes increasingly intense, which creates the entropy caused by friction due to the movement of the fluid higher.

Figure 7B stand out the influence of different values of the Reynolds and Darcy numbers on the average Bejan and Nusselt numbers, respectively.

It is seen that there is a direct relationship between Nusselt’s number and both the Darcy and Reynolds numbers, where the heat transfer is improved whenever the Darcy or Reynolds numbers are heightened. However, the principal reason behind that is, by increasing Re and Da, the fluid motion and the fluidity increase, and that enhances the heat transfer of the whole system, where the thermal energy is transmitted more efficiently as much as the convection is dominated (especially with high Reynolds values, the forced conviction is the dominated mode).

The development of the Bejan number takes two separated stages (for Re < 100 and Re > 100), in general, Be drops with increasing Re for all Darcy values. Furthermore, for Re < 100 the Bejan number declines in an important manner, where the lowest values of Be are touched as the following order: for Da = 10^–5^, 10^–2^, 10^–3^, then 10^–4^. Moreover, for Re > 100, the tendency of decrement is tiny and the lowest values of Be are reached by reducing Da from 10^–2^ to 10^–5^, where case of Da = 10^–4^ provides the lowest values. As Re increases, the velocity of the fluid in particular will be increasing, which leads the entropy (caused by fluid friction) to build up and the ratio of entropy generation (due to heat transfer over the entropy generation caused by fluid friction) to drop. In another part, it has been observed that Be increases with increasing Da, however, it might be explained that the entropy generation caused by heat transfer is heightening due to increasing Da.

**Figure 8:**
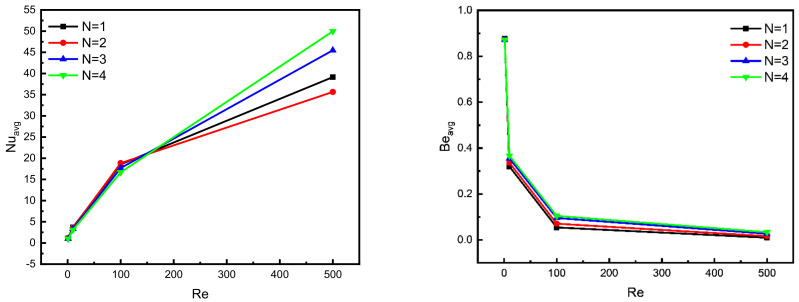
Effect of N on Nu_avg_ and Be_avg_ for φ = 0.04, Ha = 0, and Da = 10^−2^.

The effects of the peak number of the two side walls (N = 1, 2, 3, and 4) undergoing different Reynolds number values on the Nuavg and Beavg are presented in Figure 8. It is shown that Re has a direct relationship with the Nuavg and an opposite one with the Bejan number, wherewith high values of Reynolds, the active motion of the fluid is more substantial, thus, the heat would be capable of transferring more efficiently and the entropy that is generated by the friction inside the fluid during its movement would be heightened, consequently, the irreversibility decreases. On other hand, it is detected that the undulation peak number positively affects the Nusselt and heat transfer, where the influence of the undulation pattern is obvious for Re > 100, yet no remarkable influence is noted when Re < 100.

However, an odd result is detected where the heat transfer rate is low, as with the case of N = 2. The reason behind this could be that the convection flow for N = 3 and over makes the flow well mixed near the zigzagged board but for N = 2, the flow motion struggles to be mixed as well as that which occurs in higher undulations, meanwhile, the flow does not move as freely as with the case of N = 1.

Based on these remarks, it can be declared that high wall undulation boosts the heat transfer by animating the fluid motion. In addition, the Bejan number is also affected by the undulation pattern of the walls, where the lowest *Be* values are obtained by using the lowest peaks number.

## 5. Conclusions

The mixed convection mode is introduced through this work in a closed porous chamber consisting of two wavy walls, one hot and the other cold, with the rest of the walls insulated. All the enclosure walls are motionless except for the upper wall, which is in a horizontal sliding movement along the positive x-direction, to realize the mode of forced convection. The movement of the fluid, in general, is subjected to the buoyant force coming from the natural convection and the top wall motion. In addition, the utilized hybrid nanofluid is exposed to a magnetic field. The effects of the Darcy, Hartman, and Reynolds numbers, and the use of different wavy designs on the two-sided walls have been displayed and analyzed, thus, the following conclusions can be drawn:

- The flow motion is directly related to the Darcy and Reynolds numbers, as a result, both heat transfer and the Nusselt number enhance by extending these numbers.
- The magnetic field is always an undesirable factor in heat transfer, as it reduces the fluid motion, in return, the effectiveness of the convection mode is diminished.
- The undulation pattern has an active role in improving the heat distribution by increasing the fluid motion, especially near the wavy walls.
- Increasing the Darcy or Reynolds numbers builds up the entropy that is generated from fluid frictions and reduces the Bejan number in other parts.
- Despite the increased entropy production due to heat transfer by enriching the peaks of the wavy walls, the Bejan number decreases because the entropy generation caused by fluid friction is higher than the entropy caused by the heat transfer.
- The magnetic field negatively affects the fluid friction entropy, causing the entropy production resulting from the conduction heat transfer mode to be dominated, thus, the Bejan number reaches high values as the Hartman number increases.

## Figures and Tables

**Table 1:** Nanoparticles and base fluid thermophysical properties [42,43].

	Pure Water	Fe_3_O_4_	MWCNT
Cp (J/kg·k)	4179	670	710
ρ (kg/m^3^)	997.1	5180	2100
k (W/m·k)	0.613	9.7	2000

**Table 2:** The hybrid Nano-liquid thermophysical properties [42].

Properties	Correlations
Density	$\rho_{hnf}=\left(1-\varphi \right)\rho_{f}+\varphi \rho_{np}$
Heat capacity	${\left(\rho c_{p}\right)}_{hnf}=\left(1-\varphi \right){\left(\rho c_{p}\right)}_{f}+\varphi {\left(\rho c_{p}\right)}_{np}$
Thermal expansion coefficient	${\left(\rho \beta \right)}_{hnf}=\left(1-\varphi \right){\left(\rho \beta \right)}_{f}+\varphi {\left(\rho \beta \right)}_{np}$
Electrical conductivity	$\sigma_{hnf}=\left(1-\varphi \right)\sigma_{f}+\varphi \sigma_{np}$
Thermal conductivity	$k_{hnf}=\frac{k_{np}+\left(n-1\right)k_{f}-\left(n-1\right)\left(k_{f}-k_{np}\right)\varphi }{k_{np}+\left(n-1\right)k_{f}+\left(k_{f}-k_{np}\right)\varphi }k_{f}$
Viscosity	$\mu_{hnf}=\frac{\mu_{f}}{{\left(1-\varphi \right)}^{2.5}}$

**Table 3:** Different mesh sizes for Ha = 0, Ω = 0, and φ = 0.02.

Grid Elements	76837	139762	182734	480787	913657
Nu_avg_	41.214	41.723	41.809	41.804	41.804
Be_avg_	0.10362	0.10406	0.10421	0.10421	0.10420

**Table 4:** The values of the dimensionless used numbers.

Dimensionless Number	Value Range
Da	10^–2^–10^–5^
Re	1–500
Ha	0–100
Ω	−500–1000
Ra	10^5^ *

* constant value; remark: if using dimensionless numbers, the ranges are based on common academic proposals in the literature [9,10,11,12,13,14,15,16,17,18,19,20,21,22,23,24,25,26,27,28,29,30,31,32,33,34,35,36,37,38,39].

## Data Availability

The data used to support the finding of this study are included within the article.

## Nomenclatures

Symbols	
*B*	Magnetic field
*Be*	Bejan number
*c_p_*	Specific heat (J·kg^−1^ K^−1^)
*Da*	Darcy number
*g*	Gravitational acceleration, (m·s^−2^)
*Ha*	Hartman number
*k*	Thermal conductivity, (W·m^−1^·K^−1^)
*L*	Cavity width, (m)
*Nu*	Nusselt number
*P*	Dimensionless pressure
*Pr*	Prandtl number
*Ra*	Rayleigh number
*Re*	Reynolds number
*Ri*	Richardson number
*S*	Entropy generation
*T*	Temperature, (K)
*u*, *v*, *w*	Velocities in x, y, z-directions, (m·s^−1^)
*U*, *V*, *W*	Dimensionless velocities component in X, Y, Z-directions
*X*, *Y*, *Z*	Dimensionless coordinates in x, y, z directions
Greek letters	
α	Thermal diffusivity (m^2^·s^−1^)
β	Thermal expansion coefficient (K^−1^)
ε	Porosity
θ	Dimensionless temperature
κ	Permeability, (m^2^)
μ	Dynamic viscosity (Pa·s)
ν	Kinematic viscosity, (m^2^·s^−1^)
ρ	Density, (kg/m^3^)
σ	Electrical conductivity, (Ω^−1^·m^−1^)
φ	Nanoparticle concentration
Subscript	
*f*	fluid
*Ff*, *FF*	Fluid friction
*hnf*	Hybrid-nanofluid
*Mf*, *MF*	Magnetic field
*nf*	Nano-fluid
*np*	Nano-particle
*s*	Solid matrix
*hT*, *HT*	Thermal
*Tot*, *TOT*	Total
